# Super-resolution STED imaging in the inner and outer whole-mount mouse retina

**DOI:** 10.3389/fopht.2023.1126338

**Published:** 2023-04-06

**Authors:** Leon Kremers, Kseniia Sarieva, Felix Hoffmann, Zhijian Zhao, Marius Ueffing, Thomas Euler, Ivana Nikić-Spiegel, Timm Schubert

**Affiliations:** ^1^ Institute for Ophthalmic Research, University of Tübingen, Tübingen, Germany; ^2^ Werner Reichardt Centre for Integrative Neuroscience (CIN), University of Tübingen, Tübingen, Germany; ^3^ Institute for Experimental Epileptology and Cognition Research, University of Bonn, Bonn, Germany; ^4^ International Max Planck Research School for Brain and Behavior, Bonn, Germany; ^5^ Hertie Institute for Clinical Brain Research, University of Tübingen, Tübingen, Germany

**Keywords:** retina, horizontal cells, ganglion cells, diffraction limit, super-resolution, synapses, mouse, STED

## Abstract

Since its invention, super-resolution microscopy has become a popular tool for advanced imaging of biological structures, allowing visualisation of subcellular structures at a spatial scale below the diffraction limit. Thus, it is not surprising that recently, different super-resolution techniques are being applied in neuroscience, e.g. to resolve the clustering of neurotransmitter receptors and protein complex composition in presynaptic terminals. Still, the vast majority of these experiments were carried out either in cell cultures or very thin tissue sections, while there are only a few examples of super-resolution imaging in deeper layers (30 - 50 µm) of biological samples. In that context, the mammalian whole-mount retina has rarely been studied with super-resolution microscopy. Here, we aimed at establishing a stimulated-emission-depletion (STED) microscopy protocol for imaging whole-mount retina. To this end, we developed sample preparation including horizontal slicing of retinal tissue, an immunolabeling protocol with STED-compatible fluorophores and optimised the image acquisition settings. We labelled subcellular structures in somata, dendrites, and axons of retinal ganglion cells in the inner mouse retina. By measuring the full width at half maximum of the thinnest filamentous structures in our preparation, we achieved a resolution enhancement of two or higher compared to conventional confocal images. When combined with horizontal slicing of the retina, these settings allowed visualisation of putative GABAergic horizontal cell synapses in the outer retina. Taken together, we successfully established a STED protocol for reliable super-resolution imaging in the whole-mount mouse retina at depths between 30 and 50 µm, which enables investigating, for instance, protein complex composition and cytoskeletal ultrastructure at retinal synapses in health and disease.

## Introduction

**Figure 1:**
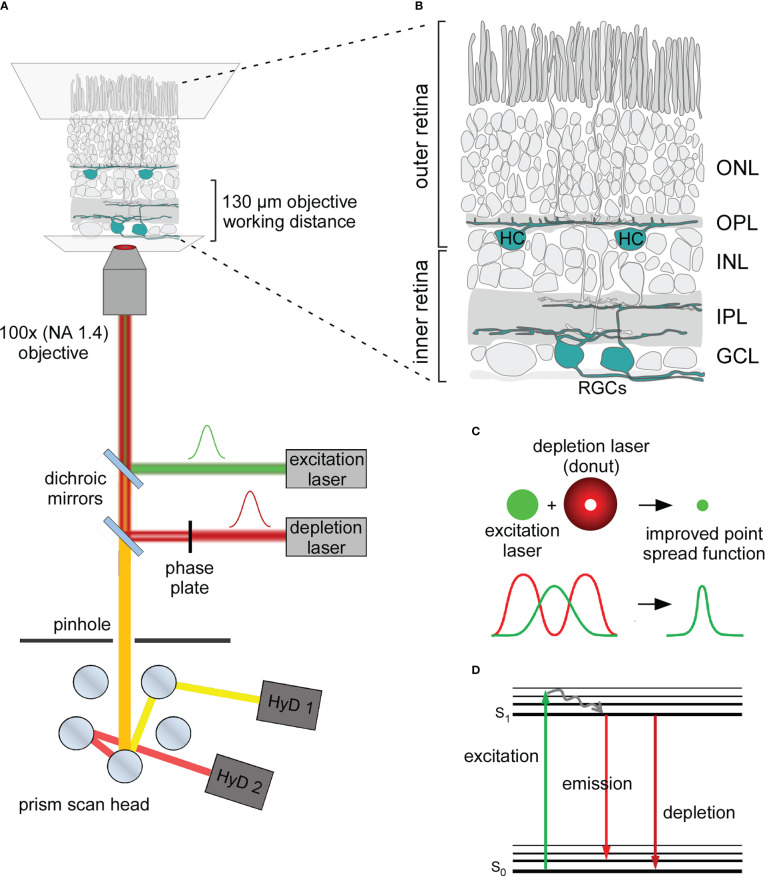
Principle of STED microscopy and application in the mouse retina **(A)** Schematic organisation of an inverted STED microscope used in this work. In addition to the excitation laser (green) the microscope includes a red shifted depletion laser (red) which is converted into a donut shape by passing through a gradient phase plate. Depletion and excitation lasers are aligned with dichroic mirrors and focused on the sample. Emission photons (orange) are passed back through a pinhole. Distinct emission wavelengths are separated in the prism scan head and transmitted to multiple hybrid detectors (HyDs). NA, numerical aperture. **(B)** Illustration of the whole-mount mouse retina for STED imaging of retinal ganglion cells (RGCs) in the inner retina and horizontal cells (HCs) in the outer retina depicted in cyan. ONL, outer nuclear layer; OPL, outer plexiform layer; INL, inner nuclear layer; IPL, inner plexiform layer; GCL, ganglion cell layer. **(C)** Lateral resolution is improved with STED microscopy: The donut-shaped depletion laser depletes emission from the periphery of the excited volume and reduces the effective point spread function. **(D)** Jablonski diagram depicting the energetic principles behind the fluorescent depletion effect. Excitation light lifts the fluorophore from its ground state (S_0_) into a higher energetical level (excitation). The fluorophore drops into the S_1_ state *via* vibrational relaxation. When the fluorophore drops from its S_1_ to its S_0_ state energy is released in the form of fluorescent emission (emission). However, when the fluorophore in its S_1_ state is depleted the energy level decreases without emitting fluorescence (depletion).

Super-resolution microscopy combines the advantages of fluorescent imaging with resolutions below the diffraction limit of light and has been abundantly used to image biological specimens (1–4). It is especially relevant for neuroscience, where important subcellular structures (e.g. synaptic structures) have sizes below the diffraction limit (5, 6). Thus, it is not surprising that different super-resolution techniques were applied, e.g., to resolve clustering of neurotransmitter receptors (7, 8) or protein complex composition in presynaptic terminals (9, 10). So far, the majority of these experiments were carried out either in cell cultures or thin tissue sections. In contrast, there are only a handful of examples of super-resolution imaging in thick specimens (11, 12), as imaging deep tissue is challenging for most super-resolution techniques (13). Specifically, the coordinate-stochastic approaches (e.g. PALM, STORM) (14–17) have more fundamental restrictions for imaging of non-superficial structures due to the total internal reflectance fluorescence (TIRF) microscopy configuration that limits fluorescence illumination to the thin layer immediately adjacent to the glass coverslip (18). In contrast, coordinate-targeted approaches, such as stimulated emission depletion (STED) microscopy, can potentially image in deeper layers (30 - 50 µm) of biological samples (e.g. the whole-mount mouse retina) by taking the advantage of optical sectioning originating from the confocal basis of the setup (19) (**Figures 1A, B**).

STED microscopy differs from confocal microscopy by including an additional “donut-shaped” depletion laser, which is aligned to the central Gaussian-shaped excitation laser (**Figures 1A–C**). Here, the fluorophore is first excited by the excitation laser, and then it is either subdued by the depletion laser or spontaneously emits fluorescence (**Figure 1D**). The donut shape of the depletion laser enables fluorescence emission from the centre while restricting it in the spatial surround. This scales the effective point spread function (PSF) down by reducing the volume from which fluorescence is generated and detected (**Figures 1C, D**) (4, 20). The main challenge when using STED imaging lies in the additional artefacts created by biological tissue: absorption, spherical aberration and light scattering intensify with increasing tissue depth (19), resulting in the generation of out-of-focus fluorescence and minimising the STED effect. To some extent, one can compensate for these effects by increasing the intensities of both excitation and depletion lasers. However, given that STED requires high laser intensities by itself, further increasing the laser power comes with a trade-off in photo-damaging effects and thermal drift as well as reducing the signal-to-noise ratio (SNR), which is already quite low in STED microscopy *per se*. Even though in-depth super-resolution imaging is challenging, there are questions in neuroscience that can be only addressed within thick specimens. For instance, STED imaging was applied in acute hippocampal slices at depths of 90–120 µm, where it helped to resolve the actin dynamics of dendritic spines (11).

As a part of the brain, the mammalian retina has a defined layered structure (21) that enables easy access to neuronal compartments. Moreover, the chemical and electrical synapse types in the retina represent those found across the rest of the central nervous system quite well (22–24). Given these features, valuable insights can be gained by applying super-resolution microscopy to the retina (25, 26).

At the surface of the retinal tissue, retinal ganglion cells (RGCs) sample the visual input *via* the vertical photoreceptor-bipolar cell pathway and project the information *via* the optic nerve to higher areas of the brain (**Figure 1B**) (27). Using immunolabeling against some neurofilament structures, specific types of RGCs can be easily visualised. This approach offers two advantages: first, it labels only a subfraction of all RGC types, and thus, enables the identification of individual cells. Second, labelling the neurofilament structures visualises all cellular compartments of an individual RGC – soma, dendrites, and axon. Therefore, neurofilament staining provides a suitable starting point for establishing a high-resolution imaging approach.

Horizontal cells (HCs) are interneurons in the outer retina. The complex feedback synapses between photoreceptors and HCs in the mouse retina are anatomically and functionally well described (28). In addition, they have been shown to form synaptic contacts with bipolar cells (BCs) using electron microscopy (29–32). A recent study has identified bulbs along HC dendrites, which likely represent the synaptic contacts between HCs and BCs (33). Intriguingly, these bulbs are located below the very distal HC dendritic tips, suggesting that they do not contact cones, but co-localize with synaptic landmarks, such as mitochondria and GABA_C_ receptors. The presence of such a HC-to-BC feedforward signalling spatially separated from the HC-photoreceptor contacts is appealing, because HC feedback to photoreceptors is thought to operate locally and to be minimally influenced by global HC computations (34). Still, HC feedforward signalling is still understudied and functional experiments testing the involvement of HC-to-BC signalling in the generation of BC responses are missing.

Taken together, the complex synaptic connectivity pattern of mouse HC cells with distinct synaptic sites (33) and synaptic mechanisms (28), makes this interneuron one of the most fascinating cells in the mouse retina. In particular, at the level of synaptic and subsynaptic organisation, HCs may always be good for a surprise (34). In this study, we developed a reliable protocol for STED imaging in the whole-mount mouse retina. First, we optimised the sample preparation and imaging settings for imaging RGC neurofilament structures close to the surface of the retinal tissue. Second, we adapted our approach for imaging synaptic structures of HCs deeper in the outer retina.

## Methods

### Animals & whole-mount retina tissue preparation

Retinae from adult (4-13 weeks old) male and female mice of the C57BL/6J wildtype line were used for this study. The animals were deeply anaesthetized with isoflurane (CP-Pharma, Germany) and were sacrificed by cervical dislocation. All animals were handled in accordance with the European and national government regulations following the European animal welfare law. The eyes were quickly enucleated, and all further dissection steps were performed in 0.1 M phosphate saline buffer (PBS) (pH 7.4). Cornea, lens and vitreous body were carefully removed. The retina was dissociated from the eyecup and mounted RGC side-up as a whole retina or cut in three or four pieces on black nitrocellulose membrane (0.8 mm pore size, Millipore, Ireland).

### Immunohistochemistry of the inner retina

The whole-mount retina preparations (for imaging of the inner retina) were fixed using 4% paraformaldehyde (PFA) in 0.1 M PBS for 20 minutes at 4°C, washed with 0.1 M PBS (6 x 20 minutes at 4°C) and blocked with blocking solution (10% normal goat serum (NGS) and 0.3% Triton X-100 in 0.1 M PBS) overnight at 4°C. Afterwards, the samples were incubated with primary antibodies (see **Table 1** below) solution with 0.3% Triton X-100 and 5% NGS in 0.1 M PBS for 4-9 days at 4°C. The samples were then washed with 0.1 M PBS (6 x 20 minutes at 4°C) and incubated with secondary antibody (see **Table 2** below) solution in 0.1 M PBS overnight at 4°C. After another washing step (6 x 20 minutes at 4°C), the retinae were embedded in mounting media on a glass slide. Different types of mounting media were used. ProLong Gold (ThermoFisher Scientific, USA) and Vectashield (Vector Laboratories, USA) were used according to the Manufacturers’ protocol. Abberior TDE Mounting Medium O (Abberior, Germany), was used according to manufacturer prescriptions with elongation of each incubation step to 1 hour (see description below). The samples were covered with high-precision coverslips (No. 1.5H, Carl-Roth GmbH, Germany), sealed with transparent nail polish and left overnight at 4°C.

**Table 1:** The following primary antibodies were used.

Target protein	Host species	Clonality	Antibody isotype	Dilution factor	Catalogue no.	Manufacturer
Neurofilament H (SMI32)	Mouse	Monoclonal	IgG1	1:100	801701	BioLegend, USA
Calbindin	Guinea pig	Polyclonal	IgG	1:500	214 005	Synaptic Systems, Germany
GABA ρ2 receptor	Rabbit	Polyclonal	IgG	1:500	AGA-007	Alomone Labs, Israel

**Table 2:** The following species-specific secondary antibodies were used.

Host species	Target species	Isotype	Conjugated dye	Dilution factor	Catalogue no.	Manufacturer
Goat	Mouse	IgG	Alexa Fluor 488	1:100	A-11001	Invitrogen Antibodies, USA
Goat	Mouse	IgG	STAR488	1:100-1000	ST488-1001-500UG	Abberior, Germany
Goat	Mouse	IgG	Chromeo 488	1:1000	15031	Active Motif, USA
Goat	Mouse	IgG	ATTO532	1:100	610-153-121	Rockland, USA
Goat	Mouse	IgM (heavy chain)	Alexa Fluor 633	1:100-400	A-21046	Invitrogen Antibodies, USA
Goat	Mouse	IgG	STAR635P	1:100	ST635P-100–1-500UG	Abberior, Germany
Goat	Mouse	IgG	ATTO647N	1:100	50185	Sigma Aldrich, USA
Goat	Rabbit	IgG	Alexa Fluor 488	1:750	A-11008	Invitrogen Antibodies, USA
Goat	Rabbit	IgG	ATTO488	1:100	18772-1ML-F	Sigma-Aldrich, USA
Goat	Rabbit	IgG	ATTO633	1:100	41176-1ML-F	Sigma-Aldrich, USA
Goat	Guinea pig	IgG	STAR488	1:100	ST488-1006-500UG	Abberior, Germany
Goat	Guinea pig	IgG	Alexa Fluor 647	1:500	106-605-003	Jackson Immuno Research, UK

### Cryosectioning and immunolabeling of horizontal sections of the outer retina

To prepare retinal pieces for horizontal sectioning (and imaging of the outer retina), retinal pieces, isolated as previously described, were fixed in 4% PFA solution for 20 minutes as described above. After fixation, the retinal pieces were washed in 0.1 M PBS (3 x 10 minutes) at 4°C, separated from the nitrocellulose membrane and passed through a series of incubations in sucrose-PBS solutions with increasing concentration. All sucrose incubations were performed at 4°C. First, retinae were kept in 10% sucrose solution for 1 to 2 h, until all pieces sank to the bottom. Retinae were then transferred into 20% sucrose solution and incubated for an additional 1 to 2 hours, again until all pieces subsided, before being transferred to a 30% sucrose solution, in which they were kept overnight. Retinal pieces were transferred into tissue freezing medium for preincubation before being mounted to the sample holders of an Epredia CryoStar NX50 Cryotome (Thermo Fisher Scientific, USA). In order to section the retinae along the xy axis as horizontally as possible, tissue freezing medium was applied to the sample holders of the cryotome and frozen solid. The cryotome was then used to cut an even plane into the frozen medium big enough to fit one retinal piece. Retinal pieces were aligned on a glass slide wrapped in parafilm with the RGC layer facing downwards. Single pieces were then picked up by carefully descending the plane of frozen medium with the sample holder on it. Additional freezing medium was used to fully cover the retina, before the sample was quickly frozen with liquid nitrogen. The sample holder was then placed back into the cryotome and 50 µm thick horizontal sections of the retina were cut. Cut sections were picked up using superfrost slides (Thermo Fisher Scientific, USA) and dried for 1 hour at 37°C on a heating plate.

Retinal slices mounted on microscopy slides were surrounded by PAP pen (Science Services, Germany) and solutions were directly applied on the slides. After cryosectioning, the retinal sections were washed in 0.1 M PBS (6 x 20 minutes) at 4°C. 0.1 M PBS was removed and unspecific antigens were blocked by incubating the retinae in 10% NGS and 0.3% Triton-X-100 in 0.1 M PBS overnight. The blocking solution was then removed, and primary antibodies were added (see **Table 1**). The antibodies were diluted in 5% NGS and 0.3% Triton-X-100 in 0.1 M PBS to the concentration specified by the respective manufacturer. To accommodate the distinct diffusion time of the antibody within the cut samples of minor thickness, retinal slices were incubated for two days. After incubation, unbound antibodies were removed by washing the retinae again (6 x 20 minutes) with 0.1 M PBS at 4°C before adding the secondary antibody. Secondary antibodies, which are conjugated to fluorophores of choice (see **Table 2**), were diluted in 0.1 M PBS to the concentration specified by the manufacturer and retinae were incubated with the solution overnight. To remove excess antibodies, the retinae were again washed in 0.1 M PBS (6 x 20 minutes) at 4°C. All retinae were mounted with a 2,2′-thiodiethanol-based embedding medium (TDE, Abberior, Germany. Some retinae were mounted with the 120 µm thick spacers (Secure-seal spacer, Invitrogen, USA) between slice and coverslip. One drop of 10% TDE solution was added to cover the retinal pieces and left to incubate for 1 hour at 4°C. Afterwards, the 10% solution was exchanged with 25% solution and incubated again for 1 hour, before being exchanged again for a 39% TDE solution. The retinae were incubated in the 39% solution for 45 min before the medium was substituted for the final 97% TDE solution. The samples were left to incubate for an additional 45 minutes before and high-precision coverslips were carefully put on top of the retinal pieces and spacers. The sample was sealed by applying nail polish to the edges of the coverslip and the polish was left to dry overnight at 4°C before imaging experiments were performed.

### Confocal and STED imaging

Confocal and STED imaging were both performed at the same microscope setup using a DMi8 inverse microscope (Leica, Germany) with three oil immersion objectives with 20x (NA 0.75), 63x (NA 1.4) and 100x (NA 1.4) magnification (Leica, Germany) in combination with the TCS SP8 STED setup (Leica, Germany). The microscopy setup included three pulsed excitation lasers, one with 488 nm (Leica, Germany) and two additional lasers with 532 nm and 635 nm wavelengths (OneFive, Switzerland). For depletion, a continuous-wave 592 nm and a gated/pulsed 775 nm STED laser were used. The intensities of the depletion lasers were experimentally chosen and were 0.65 W (43% of maximum value) for the 592 nm laser and 0.45 W (30% of maximum value) for the 775 nm laser. The microscope function was controlled was controlled with the LAS X software (Leica, Germany). Fluorescent emission was split and quantified using the integrated prism scan head and photomultiplier tubes (PMTs) and/or hybrid detectors (HyDs). For STED imaging only HyDs were used. The scan head allowed for the free selection of the emission wavelength spectra to be captured and measured. For STED imaging, xy-pixel and z-step size were chosen in accordance with the Nyquist–Shannon sampling theorem. This meant that STED imaging was only performed with the 100x objective and using an image size of 2048 x 2048 pixels. An additional 3-4 x zoom was applied, resulting in an effective pixel size of 14 - 20 x 14 - 20 nm. Minimal z-step size was manually calculated and set to 130 nm. Bidirectional scanning was enabled, and each line was scanned three times with pixel intensities being accumulated. For each z-section of an image stack three lines or frames were imaged and intensity values averaged. Excitation and depletion laser intensities as well as PMT/HyD gain were determined *via* testing of signal strength and photobleaching. Laser intensities and gain thus differed on a case-to-case basis but were kept consistent within experiments. Confocal imaging was performed by disabling the depletion lasers while keeping the excitation lasers on. If the same region was imaged with both STED and confocal microscopy, confocal imaging was typically performed before STED to prevent photobleaching from the high intensity depletion laser. When the same region was imaged in multiple fluorescent channels, fluorophores with higher excitation/emission wavelengths were imaged first. Imaging data was saved as lif-files, with z-stacks being represented as different series within one file.

### Image processing and analysis

Acquired images were processed and analysed by LAS X (Leica Microsystems) and ImageJ (National Institutes of Health, USA) software. Stabilisation and deconvolution of STED images were performed by Huygens Software (Version 17.10.0p6 64b, SVI, Netherlands). The deconvolution was performed with the Classical Maximum Likelihood Estimation (CMLE) algorithm under experimentally defined settings. Images were deconvoluted using theoretical PSFs calculated for the DMi8 STED microscope and the 100x (NA 1.4) objective used in this work. No lateral drift and bleaching corrections were performed, and most settings were kept at default values. The background level was estimated by software, the quality threshold was 0.001, the number of iterations was 50, the SNR was set to 7 for STED images. In ImageJ, the original and deconvolved z-stacks were typically transformed into a single maximum intensity projection and saved as raw files for further analysis. For presentational purposes image contrast and brightness were automatically optimised, channels depicted in defined colours and scale bars inserted. All quantifications and processing were performed in unprocessed (= not deconvolved or brightness-adjusted data) unless otherwise specified.

Image analysis was performed using the open-source software ImageJ in its Fiji distribution and custom scripts written in the R programming language. For direct extraction of intensity values, a line selection was performed in ImageJ and values along the selection were extracted for all channels manually or using a custom written ImageJ macro. For determination of the full width at half maximum (FWHM), line profiles were fitted (Gaussian curve, non-linear least square (NLS) approximation) using R free programming software. In short, raw image files were imported into R and transformed into intensity value matrices. Coordinates were selected using the shiny plug-in for R and intensity values along a vector between both coordinates were saved. A Gaussian curve was fitted to these values using the NLS approach and the nls-multstart package. The general formula for the fitted curve was defined as:


$$f\left(x\right)\;=\;k\times e^{-\frac{{\left(x-u\right)}^{2}}{2s^{2}}}+m$$

The x values with $f\left(x\right)\;=\;m\;+\;0.5\times \left(f_{max}-\;m\right)$ were defined and the distance between both x values was calculated as the FWHM: For structures in the inner retina (RGC structures), we used an unconstrained NLS fit. In the outer retina (HC structures and GABA receptors), we used multiple constraints in our model: First, we defined *s* as *s* ≥ 25 determining the width of the Gaussian curve with a minimal FWHM of 58.8 nm. With our imaging system, sample probes and previous results from the RGC layer we did not expect FWHM values below this limit. Second, under the assumption that the brightest pixels along the line represent the structure we are interested in, we defined *f_max_* ≥ maximum intensity, and thus, prevented the model from being biassed by unspecific background noise. Third, we defined *u* as 50 ≤ *u* ≤ length of line -50 nm to ensure that the model doesn’t fit the curve to unspecific noise at the borders of the extracted vector. Overall, constraining our model produced fits that improved in describing the observed signal. The process was repeated with the previously defined coordinates for all corresponding images. It has to be emphasized that the measured FWHMs depend strongly on the structures measured and only give an approximation of the spatial resolution that can be achieved in this sample. The smallest measured FWHM that we could achieve in xy with this STED microscope (as measured with 100 nm fluorescent beads) was 106.8 ± 4.1 nm (mean ± SD, n = 2) nm with the 532 nm excitation/775 nm depletion laser pair. However, we expect that smaller fluorescent beads/quantum dots would allow us to measure smaller FWHMs and get a better estimate of the best resolution.

For the resolution enhancement factor (REF), the ratios between corresponding confocal and STED FWHMs as well as STED and deconvolved STED FWHMs were calculated and saved along the absolute FWHMs. Furthermore, the formulae of the fitted curves, the extracted intensity values and the determined coordinates were also saved in the same Excel (.xlsx) file.

For calculating the theoretical REF ( $REF^{th}$ ) the following formula was applied:


$$REF^{th}\;=\;\frac{d_{conf}^{th}}{d_{STED}^{th}}\;=\;\frac{0.61\;\;\times \;\lambda }{nsin\alpha }/\frac{\lambda }{2nsin\alpha \sqrt{1\;+\;a\frac{Imax}{Is}}}$$

The signal-to-background ratio (SBR) was calculated as $\frac{I_{signal}}{I_{background}}$ ,where $I_{signal}$ was defined as the maximum pixel intensity along the previously defined line selection and $I_{background}$ as the term m from the fitted Gaussain curve. We omitted values where $I_{background}\leq \;1$ as we believe that these backgrounds were unreasonably dark.

For dendritic bulb identification, pixel intensities for the Calbindin and SMI32 stainings were normalised in ImageJ. Here the brightest pixel of a z-slice was set to 255 and the darkest to 0. All other pixel intensities were scaled accordingly. The normalised pixel intensities of Calbindin and SMI32 stainings were then subtracted for a line defined across the bulb/non-bulb.

### Statistics

Data representation and statistical analysis was performed using Prism 9 software (GraphPad, US) or in the R programming language. For Prism 9, data sets were copied into grouped tables as required and statistics were calculated using the analysis function. For complex data featuring various groups, each with multiple subcategories, 2-way ANOVA combined with a Tukey’s multiple comparisons test was performed. Paired data with only two categories was subjected to Shapiro-Wilk normality testing and two-tailed paired t-test was used for analysis of normally distributed data, Wilcoxon signed-rank test was used otherwise. Unpaired data with only two categories was analysed using an unpaired t-test or Wilcoxon rank sum test. Significance was defined as: p > 0.05 = ns, p< 0.05 = *, p< 0.01 = **, p< 0.001 = ***, p< 0.0001 = ****, p< 0.00001 = *****, p< 0.000001 = ******. Mean values in text and figures are given as mean ± standard deviation (SD).

## Results

To establish STED microscopy (**Figures 1A, B**) in the mouse retina, we used whole-mount preparations labelled with a primary antibody against neurofilament H (from here on: SMI32 labelling) and secondary antibodies conjugated with synthetic dyes. The required labelling density was achieved by increasing the concentrations of both primary (2x, **Table 1**) and secondary (≤ 10x, **Table 2**) antibodies compared to commonly used concentrations of respective antibodies. We improved the sample preparation by choosing the best-performing spacer type between slide and coverslip, mounting medium, and synthetic dyes (see Methods). We tried both conventional dyes (Alexa Fluor) and new-generation dyes (STAR, ATTO, Chromeo) specifically developed for STED microscopy. We tested photostability and selected dyes with the lowest bleaching effects at 488 and 635 nm excitation. For both green and far-red dyes, new-generation dyes were more photostable than Alexa Fluor dyes. For far-red dyes (ATTO647N and STAR635P), we never observed bleaching with our experimental conditions.

To avoid physical squeezing of the retinal tissue upon mounting, we placed spacers in between the slide and coverslip. For this purpose, we used commercially available silicon spacers.

The choice of mounting medium was determined by its refractive index (n). We tested four different mounting media: Abberior Liquid AntiFade (n = 1.38), Vectashield (n = 1.47), Abberior TDE (n = 1.51) and ProLong Gold (n = 1.47). Abberior Liquid AntiFade had a lower refractive index than the immersion oil (n = 1.52) and was thus excluded from further experiments. We hypothesised that ProLong Gold should be the most stable medium as it was the only polymerizing mounting medium in our study. However, we observed strong axial drift when switching from confocal to STED mode, which we could not correct for. Vectashield mounting medium is not recommended by STED manufacturers because it absorbs light in the red range of the spectrum and is incompatible with large-Stokes shift dyes. However, in our experiments, only a minor difference in resolution with Vectashield and Abberior TDE could be observed (resolution of STED images determined as the full width at half maximum (FWHM, see Methods); with Abberior TDE: 118.2 ± 23.2 nm (n = 24 structures, n = 3 images); with Vectashield: 144.9 ± 35.1 nm (n = 20 structures, n = 3 images; mean ± SD, p = 0.01, Wilcoxon rank sum test). Finally, we chose Abberior TDE as the most stably performing mounting medium with its only constraint being the short lifetime of the specimens at around 1.5 - 2 weeks in our hands.

### Optimization of acquisition settings for STED microscopy

**Figure 2:**
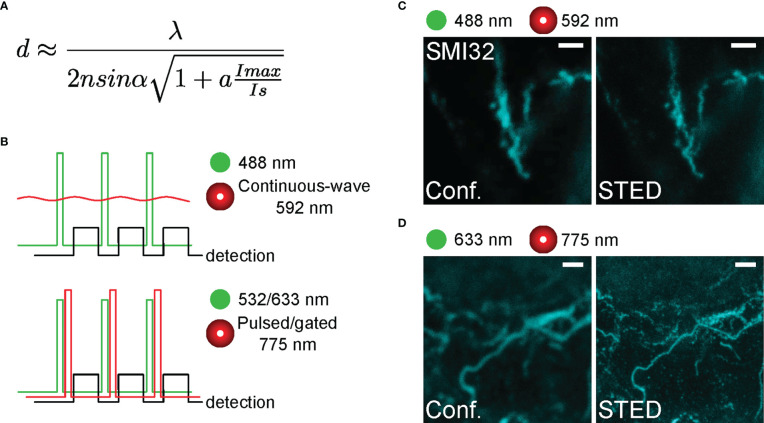
Representative SMI32-labelled structures in the inner retina acquired with different STED lasers **(A)** Formula for the lateral resolution of STED microscope with the saturation factor I_max_/I_s_ with I_max_ as the maximally applied laser intensity and I_s_ as the STED laser intensity at which half of the fluorescence is lost. **(B)** Temporal conditions of STED imaging. Ideally, all depleting photons act when fluorophores are in the singlet-excited state S_1_ and fluorescence must be registered after the stimulating photon’s action. Experimental time sequences are shown for the excitation (green), the depletion (red), and the emission signal detection (black) for continuous-wave STED (592 nm, top) and gated/pulsed STED (775 nm, bottom) microscopy. **(C, D)** Representative confocal (left) and STED (right) images acquired with different excitation and STED depletion lasers (592 nm in C, and 775 nm in D). For C, the excitation wavelength was 488 nm, and the dye was STAR488. For D, the excitation wavelength was 635 nm, and the dye was ATTO647N. Scale bars: 1 µm.

The main feature of a scanning STED microscope is the depletion laser (**Figures 1C, D**). Our STED microscope was equipped with two depletion laser lines with 592 and 775 nm wavelengths. While the 592 nm depletion laser was used together with the 488 nm excitation laser, the 775 nm depletion laser depleted the emission of fluorophores excited with either the 532 or 633 nm excitation laser. The orange beam (592 nm) is a continuous-wave laser, whereas the far-red one (775 nm) is a pulsed/gated laser (**Figure 2B**). The resolution of STED imaging depends – among other factors – on the saturation factor (I_max_/I_s_, **Figure 2A**) (35). The saturation factors for our acquisition settings were calculated by the LAS X software. With the 592 nm continuous-wave depletion laser, we could achieve a maximum saturation factor of 7.5 without severe bleaching of the 488 nm dye at laser intensity 0.65 W (**Figure 2C**). In contrast, with the 775 nm laser the saturation factors were as high as 30 for the 633 nm excitation laser (**Figure 2D**) and 28.6 for the 532 nm excitation laser at a relatively low laser intensity of 0.45 W, likely due to the fact, that a high density of 775 nm photons is ‘pumped’ into the pulsed events whereas the photon number in the between-pulse intervals is minimal (**Figure 2B**). Therefore, we suggest that using the pulsed depletion laser results in both better resolution and minor photo-damaging compared with the continuous wave depletion laser.

In general, we obtained more comparable fluorescence intensities by adjusting the excitation laser intensity for every specimen separately and increasing it with larger imaging depths. We defined the xy-pixel size as 14 - 20 nm according to the Nyquist-Shannon sampling theorem. Other imaging settings were adjusted experimentally. The bit depth was 12 bit, and we used 3x line accumulation with or without 3x frame averaging. Frame averaging was commonly used to decrease unspecific noise, however, in our case, the laser exposure was sometimes too high and led to thermal effects and physical deterioration of the sample upon imaging from the same focal plane.

### Super-resolution microscopy of retinal ganglion cell structures in the inner retina

**Figure 3:**
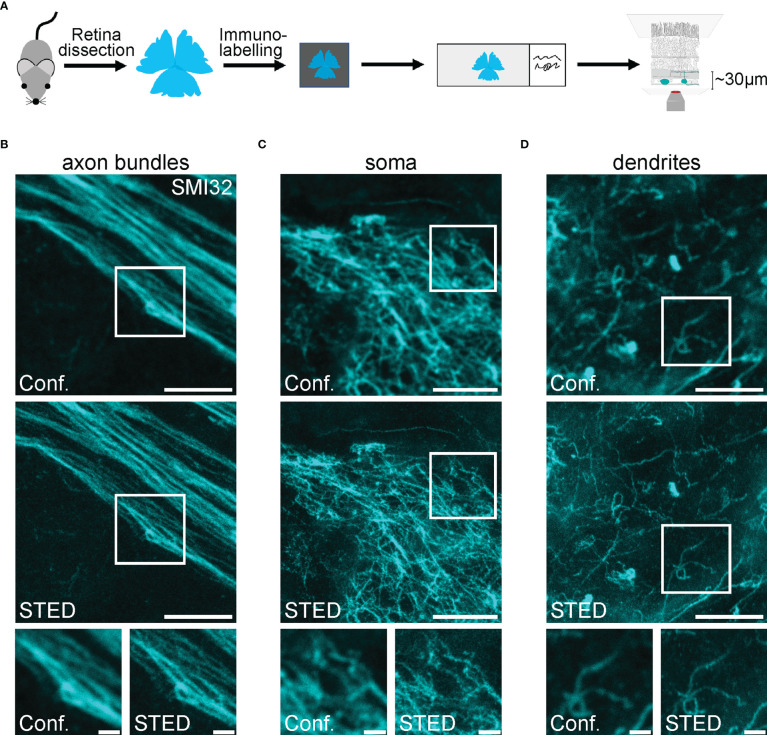
Confocal and STED imaging of SMI32-positive retinal ganglion cell structures in the inner retina **(A)** Scheme of experimental design for imaging RGC structures in the inner retina. Retinae were dissected, mounted on filter paper, immunolabelled, mounted on glass slides and imaged. **(B-D)**. Representative example images of RGCs’ axon bundles **(B)**, soma **(C)** and dendrites **(D)** imaged in the confocal (conf.) and STED mode at a depth of ~30 µm (axons, soma) and ~50 µm (dendrites). For B, the excitation wavelength was 635 nm, and the dye was STAR635P. For C and D, the excitation wavelength was 635 nm, and the dye was ATTO647N. Scale bars: 5 µm; for insets: 1 µm.

Super-resolution STED microscopy at larger depths in samples remains challenging due to scattering within biological tissue, which leads to decreased depletion efficiency in deeper layers. This restricts efficient STED imaging to the superficial 50-70 µm of the sample. In the retina this, corresponds to the ganglion cell layer with the somata and axons of RGCs, and the inner plexiform layer, which roughly comprises of dendrites and synaptic connections of RGCs, BCs and amacrine cells (ACs) (**Figures 1B**, **3A**). SMI32 labelling reveals intermediate filaments in axonal bundles of RGCs (**Figure 3B**) as well as dense cytoskeletal network structures in RGC somata (**Figure 3C**) at a depth of 25-30 µm. Imaging in the inner plexiform layer at a depth of 40-50 µm visualised the dendritic arborisation of RGCs (**Figure 3D**). However, as discussed above, the signal-to-background ratio (SBR) drops when increasing the imaging depth.

**Figure 4:**
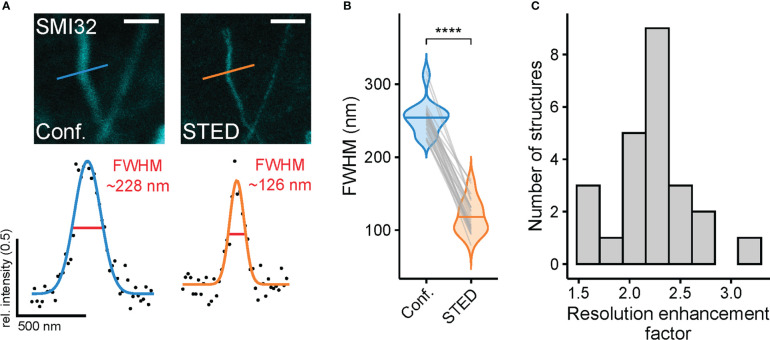
Super-resolution STED imaging of SMI32-positive retinal ganglion cell dendrites in the inner retina reveals spatial resolution enhancement **(A)** Example structures (top, dendrite of a SMI32-positive RGC) for resolution estimation and with FWHMs for dendritic structure for confocal (blue) and STED (orange) (bottom). FWHM intensity profiles taken at the indicated position in example confocal and STED images. **(B)** Comparison of FWHMs in confocal (blue) and STED (orange) images (p< 0.0001 = ****, Wilcoxon signed-rank test, n = 1 animal; n = 24 filamentous structures from 3 images, horizontal bars indicate means, grey lines connect corresponding values). **(C)** Histogram of resolution enhancement factor quantified as ratio between the FWMHs of confocal and STED images (n = 24). For A, the excitation wavelength was 635 nm, and the dye was ATTO647N. Scale bar: 1 µm in A.

As an approximation of spatial resolution, the FWHM was calculated by selecting thin filamentous structures and fitting a Gaussian curve to the intensity values of respective confocal and STED images using a custom-written R script (see Methods). The described approach allowed paired comparison between the resolution of confocal and STED images (**Figure 4A**). The mean FWHM of filamentous structures in confocal images was 254.1 ± 23.0 nm, coming close to the diffraction limit of approx. 200 nm. In comparison, the FWHM of STED images was 118.2 ± 23.2 nm and therefore surpassed the theoretical diffraction limit (n = 24 structures measured, n = 3 images, mean ± SD) (**Figure 4B**). As the FWHM is calculated from differently sized biological structures, it strongly depends on the structures selected and can vary between conditions. Thus, it does not strictly represent the theoretical maximal resolution of the microscope (see Methods). To correct for this effect, a resolution enhancement factor (REF) was calculated (35), which was defined as the ratio of STED FWHM to confocal FWHM of the same structure. Here, the REF peaked at 2.21 ± 0.36 (mean ± SD) and ranged from 1.50 to 3.09 in the dendrites of RGCs (**Figure 4C**). The maximal theoretical REF for a saturation factor 30 for this experiment is 6.79 (see Methods).

**Figure 5:**
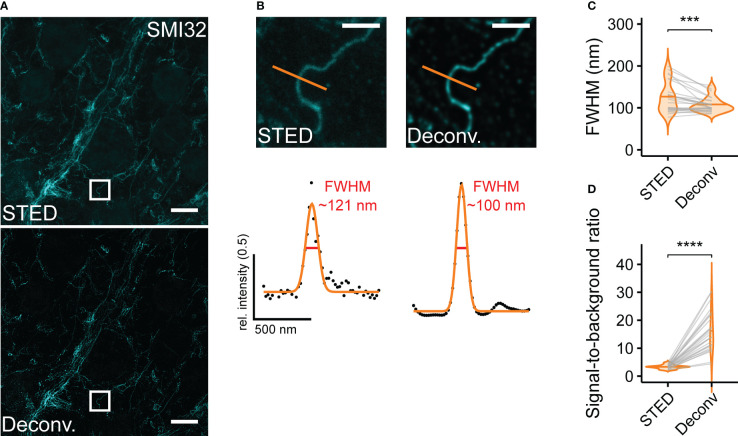
Deconvolution increases resolution and SBR for retinal ganglion cell dendrites **(A)** Representative example STED image without (top) and with deconvolution (bottom). **(B)** Top: Zoomed-in region as indicated in **(A)** Bottom: FWHM intensity profiles taken at indicated position without (STED) and with deconvolution (Deconv.) **(C)** Quantification of the effect of deconvolution on FWHMs (p< 0.001 = ***, Wilcoxon signed-rank test, n = 1 animal; n = 26 structures from 1 image, horizontal bars indicate means, grey lines connect corresponding values). **(D)** Quantification of the effect of deconvolution on SBR (p< 0.0001 = ****, paired t-test, n = 1 animal; n = 26 structures from 1 image, horizontal bars indicate means, grey lines connect corresponding values). For A and B, the excitation wavelength was 635 nm, and the dye was ATTO647N. Scale bars: 5 µm in A, 1 µm in B.

In some retinal samples, lateral and axial drift occurred at the stage of sample imaging. We tackled this problem using the Huygens Software (SVI) where appropriate. Although the image stabilisation tool performed well in lateral (xy) direction, allowing us to obtain good 3D stacks and time series, it provided no satisfying solution for axial drift (along the z-axis). The same software was used for image deconvolution (**Figure 5A**). We used a Classical Maximum Likelihood Estimation algorithm and adapted the program settings to obtain reliable deconvolution results. We compared both the FWHM and the SBR of STED and deconvolved STED images. While the deconvolution only marginally (though significantly) increased the resolution of STED images (STED, FWHM = 125.7 ± 36.0 nm; STED deconvolved, 107.2 ± 17.6 nm, n = 26 structures, mean ± SD, p< 0.001, Wilcoxon signed-rank test) (**Figures 5B, C**), it did increase the SBR (STED, 3.3 ± 0.7; STED deconvolved, 16.2 ± 7.2, n = 26 structures, mean ± SD, p< 0.0001, paired t-test) and smoothened intensity profiles of given structures (**Figures 5B–D**).

### Super-resolution microscopy of horizontal cell axon terminals in the outer retina

**Figure 6:**
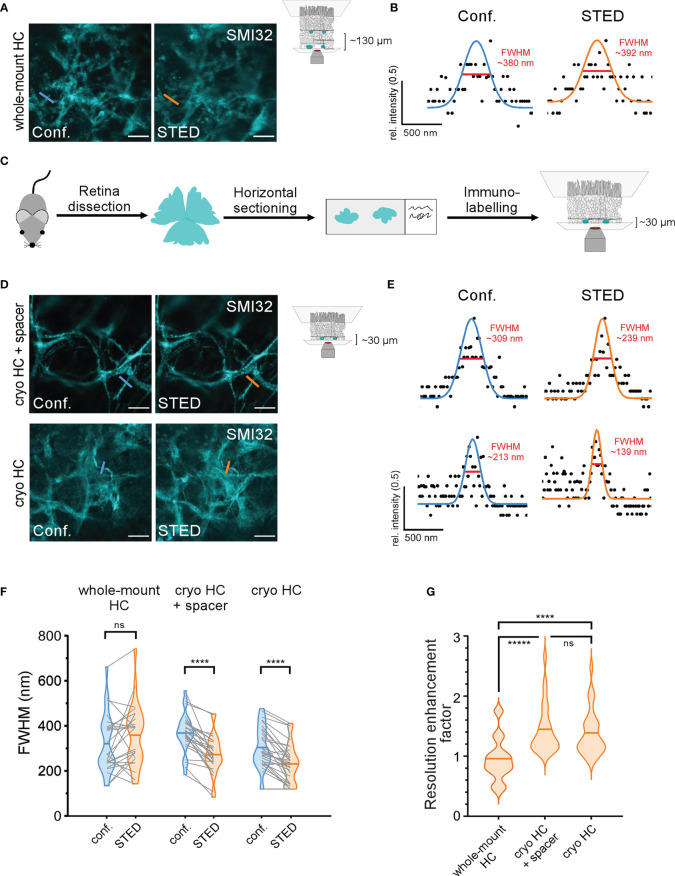
STED imaging of horizontal cell structures in the outer retina **(A)** Comparison of confocal (left) and STED (right) images of SMI32-stained HC axon terminals at a depth of ~130 µm in the retinal whole-mount imaged through the inner retina. Each image was first taken in confocal mode and then in the STED mode to allow a direct comparison. **(B)** Confocal (blue) and STED (orange) FWHMs of the same neuritic structure as indicated in **(A)**, **(C)** Scheme of alternative experimental design for imaging structures in the deeper outer retina. Retinae were horizontally cryosectioned, mounted on glass slides, immunolabelled, and imaged. **(D)** Example confocal and STED images of SMI32-stained HC axonal structures taken with (top) and without (bottom) silicon spacers between slide and coverslip. **(E)** Confocal and STED FWHMs of the same neuritic structures in **(D)**, **(F)** Quantification of FWHMs of corresponding HC structures in confocal (blue) and STED (orange) imaging mode for whole-mount condition (whole-mount HC) and horizontally cryosectioned retina (cryo HC) with and without spacers (whole-mount HC n = 1 animal/24 structures, Cryo HC + spacer n = 1 animal/26 structures, Cryo HC n = 1 animal/30 structures, p< 0.0001 = ****, ns = not significant, paired t-tests, horizontal bars indicate means, grey lines connect corresponding values). **(G)** Violin plot showing the resolution enhancement factor calculated as the ratio of the corresponding FWHMs in confocal and STED images for the three experimental conditions, horizontal bars indicate means (p< 0.0001 = ****, p< 0.00001 = *****, ns = not significant, Wilcoxon rank sum test). For A and D, the excitation wavelength was 532 nm, and the dye was ATTO532. Scale bars: 5 µm in A,D.

Compared with the inner retinal structures, we did not achieve an increase in resolution when we imaged SMI32-labelled HC axon terminals in the STED mode at larger depth in the outer retina (**Figures 6A, B, F, G**). One possible reason for this is scattering of both the excitation and depletion lasers during their passage through the tissue and, thus, misalignment of the spatially optimal laser configuration and a decrease of the STED effect. Theoretically, adaptive optics may allow the application of STED in deeper tissue while retaining super-resolution (36, 37). However, this approach would likely increase imaging duration and photobleaching. A more straightforward way, avoiding adaptive optics, is physical horizontal sectioning of the retinal whole-mount so that the structures of interest lay just below/at the section surface (**Figure 6C**, see Methods). We chose horizontal cryotome sectioning due to its widespread availability. Here, we cut off the inner layers of the whole-mounted retina to expose HC structures to the surface and image them (**Figure 6C**).

Intact and horizontally cryosectioned whole-mounted retinae were stained against SMI32 using ATTO532 as the fluorophore. One set of horizontal cryosections was surrounded by a 120 μm thick silicon spacer to minimise the physical pressure of the coverslip on the retina, to avoid squeezing and an eventual change of the FWHM of fine structures. In the other set, the retinal sections were directly touching the coverslip without any spacer (**Figures 6D, E**). Images were taken in each condition first using confocal mode and then switching to STED, and thus, imaging the exact same region. Again, we calculated the FWHMs of filamentous structures as a measure for the resolution in both confocal and STED images (**Figures 6B, E**). We did not find any significant change of the FWHM for STED compared to confocal images of HC structures in the intact retina (confocal, 319.5 ± 131.6 nm; STED, 358.3 ± 138.9 nm; mean ± SD; p = 0.11, paired t-test) (**Figures 6A, B, F**). Interestingly, in the intact whole-mount, the variability of FWHMs for STED images was strongly increased at the HC level (**Figure 6F**), likely illustrating the random effect of scattering in heterogeneous and deep tissue. In contrast, in both cryosection conditions (with and without spacers), we did not see such a pronounced effect on the variability. However, here we found significantly improved STED FWHMs with spacer (confocal 367.4 ± 86.4 nm; STED, 271.6 ± 85.6 nm; mean ± SD; p< 0.0001, paired t-test) and without spacer (confocal, 302.7 ± 94.0 nm; STED, 230.1 ± 85.4 nm; mean ± SD; p< 0.0001, paired t-test), thus indicating that horizontal sectioning indeed reduces aberrations in STED microscopy (**Figures 6D–F**). While not significantly different (confocal p = 0.09 and STED p = 0.44, 2-way ANOVA test), both confocal and STED FWHMs tended to be slightly smaller in cryosections mounted without silicon spacers, possibly reflecting a better fit with the working distance of the microscope objective. To correct for differently sized biological structures, we calculated the REF in all conditions (**Figure 6G**), which validated the previous results: STED in whole-mounted retinae did not alter the resolution in HC axon terminals (0.95 ± 0.35, mean ± SD). In contrast, the relative resolution in the cryosectioned groups was improved by the factors 1.44 ± 0.08 (mean ± SD; with spacer) and 1.38 ± 0.07 (mean ± SD; with spacer). With the saturation factor of 28.6 in this experiment, the maximal theoretical REF is 6.6. The REF in HC structures in cryosectioned retinae did not significantly differ from the whole-mounted RGC axons imaged in the same set of experiments (REF: 1.41 ± 0.50, n = 1 animal, n = 23 RGC structures; mean ± SD; p = 0.46 compared to HC with spacer and p = 0.77 compared to HC without spacer, Wilcoxon rank sum test). Interestingly, the mean SBRs of SMI32-positive HC structures were similar in the intact whole-mounted and both cryosectioned conditions (whole-mounted, 8.8 ± 15.3, n = 24 structures; cryosectioned with spacer, 12.6 ± 20.4, n = 25 structures; cryosectioned without spacer, 7.5 ± 8.6, n = 28 structures, all imaged in the STED mode, mean ± SD). However, as expected from the diffuse nature of SMI32-staining, there were large differences between the SBRs of single structures (**Figure 6D**), as indicated by the high SD values. Structures with low SBR were frequently encountered in all three conditions.

### Identification of dendritic bulbs of horizontal cells

After confirming that the antibody staining and STED imaging protocols work as anticipated for the outer mouse retina, we applied our approach to the outer synaptic circuits. Bulb structures on HC dendrites have been recently identified as putative synaptic sites between HCs and BCs (33). Therefore, we aimed at investigating this synapse type using super-resolution microscopy. We first identified HC bulb structures in the cryosectioned retina using confocal microscopy. The calcium-binding protein Calbindin is expressed throughout HCs, with anti-Calbindin staining being used to visualise the whole cell including soma, dendrites, and axon terminals. In HCs, neurofilaments are only expressed in the axon terminal system and can thus be used to distinguish between dendritic and axonal structures (38). GABA ρ2 is a subunit of GABA_C_ receptors, which in the outer retina is exclusively expressed on BCs, thus indicating postsynaptic BC sites. Before performing a triple staining, primary antibody functionality was tested in single and double stainings, observing structures known to express the targets of the antibodies with confocal microscopy (Calbindin for HC somata, dendrites and axons, SMI32 for HC axons, GABA ρ2 for GABA ρ2-expressing receptor clusters in the HC layer).

**Figure 7:**
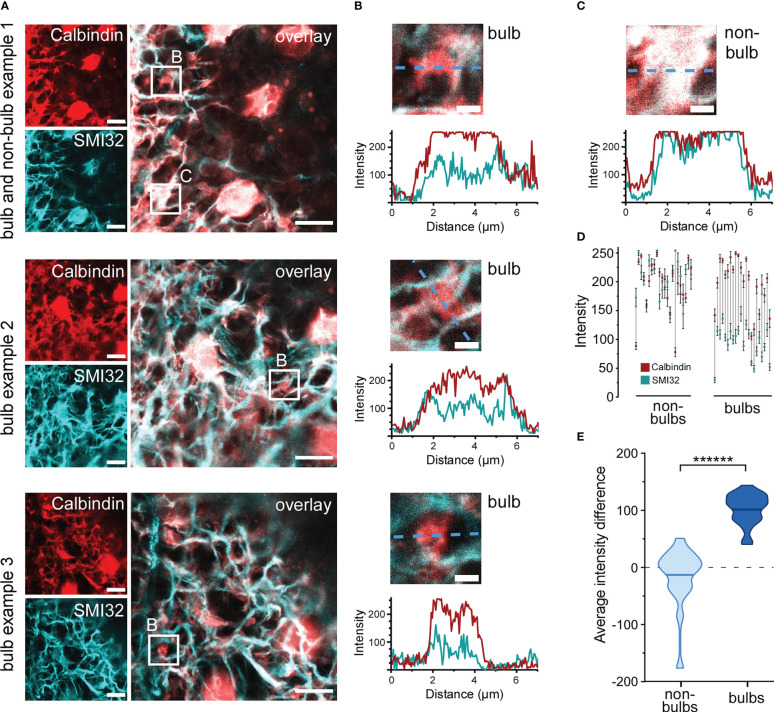
Identification of dendritic horizontal cell bulbs **(A)** Three example images showing three HC bulbs and one non-bulb identified in confocal single plane images z-stack slices. Retinal sections were imaged for Calbindin and SMI32. Bulb structures (bulb) positive for only Calbindin and non-bulb structure (non-bulb) positive for both SMI32 and Calbindin were identified (small white squares). **(B)** Intensity values for Calbindin and SMI32 stainings were extracted along a line through the three bulbs taken from **(A)** Plots show Calbindin and SMI32 intensity distribution across the three bulbs (dashed blue lines). **(C)** Intensity values for Calbindin and SMI32 stainings of a dendritic non-bulb taken from **(A)** (top panel). Plot shows Calbindin and SMI32 intensity distribution across the non-bulb (blue dashed line). **(D)** Mean intensities of Calbindin (red) and SMI32 (cyan) stainings along the lines through individual non-bulbs (left) and bulbs (right). Error bars indicate 95% confidence interval. The vertical grey lines connect corresponding Calbindin and SMI32 intensities within the same dendritic structures. **(E)** Violin plot showing the average intensity difference. Difference per pixel was calculated for multiple bulbs and non-bulb control structures, horizontal bars indicate means (n = 1 animal, n = 22 structures for bulbs, n = 22 structures for non-bulbs, p< 0.000001 = ******, Wilcoxon rank sum test). For A and B, the excitation wavelength was 532 nm, and the dye was ATTO532 for SMI32. For Calbindin, the excitation wavelength was 488 nm, and the dye was STAR488. Scale bars: 10 µm in A, 2 µm in B,C.

Dendritic HC bulbs were characterised as Calbindin-positive and SMI32-negative structures that possibly co-localize with GABA ρ2 (33). Bulb identification was performed using lower magnification confocal microscopy (100x objective, 2.1× digital zoom) and imaging Calbindin and SMI32 in large z-stacks, spanning the whole HC layer. Calbindin and SMI32 images were superimposed, and single z-slices were manually scanned for bulb structures (see examples in **Figure 7A**). Surprisingly, only few SMI32-negative structures could be observed, suggesting that HC dendrites are not isolated from axons but co-fasciculate, and thus, cannot be easily distinguished under the microscope. However, several round and ‘blobby’ SMI32-negative thickenings could still be detected emerging from double positive structures, which we assumed to be HC dendritic bulbs (**Figure 7A**). Such identified bulbs were further examined by extracting normalised SMI32 and Calbindin intensity values along a line selection which was put through each bulb (**Figures 7B, C**). Bulbs were more positive for Calbindin than for SMI32 and underlying SMI32 signals did not follow the shape of Calbindin signals (**Figure 7B**). Intensity values were also extracted from line selections of control structures (dendritic thickenings, non-bulbs), which we assumed to be double-positive (**Figure 7A** top panel, **C**). In some rare cases Calbindin or SMI32 staining intensities reached a plateau which was likely caused by signal saturation (**Figure 7B** top panel, **C**). However, within-bulb differences between the weaker SMI2 and stronger Calbindin signals could still be observed (theoretically, the signal difference would have been even more prominent without saturation) (**Figure 7D**). For further comparison and statistical analysis, mean staining intensities along the lines were calculated for each structure. SMI32 and Calbindin signals did strongly correlate in many non-bulb controls but never in the bulbs (**Figure 7D**). Indeed, the average intensity difference was significantly higher for the HC bulbs than for the non-bulb structures (bulbs, 102.55 ± 27.3; non-bulbs, -12.5 ± 47.8; mean ± SD; p< 0.000001, Wilcoxon rank sum test) (**Figure 7E**). Thus, although SMI32-positive/Calbindin-positive axons and SMI32-negative/Calbindin-positive dendrites of HCs did strongly co-fasciculate, the reliable identification of dendritic bulbs was possible.

### Identification of putative GABAergic synapse at bulb sites

**Figure 8:**
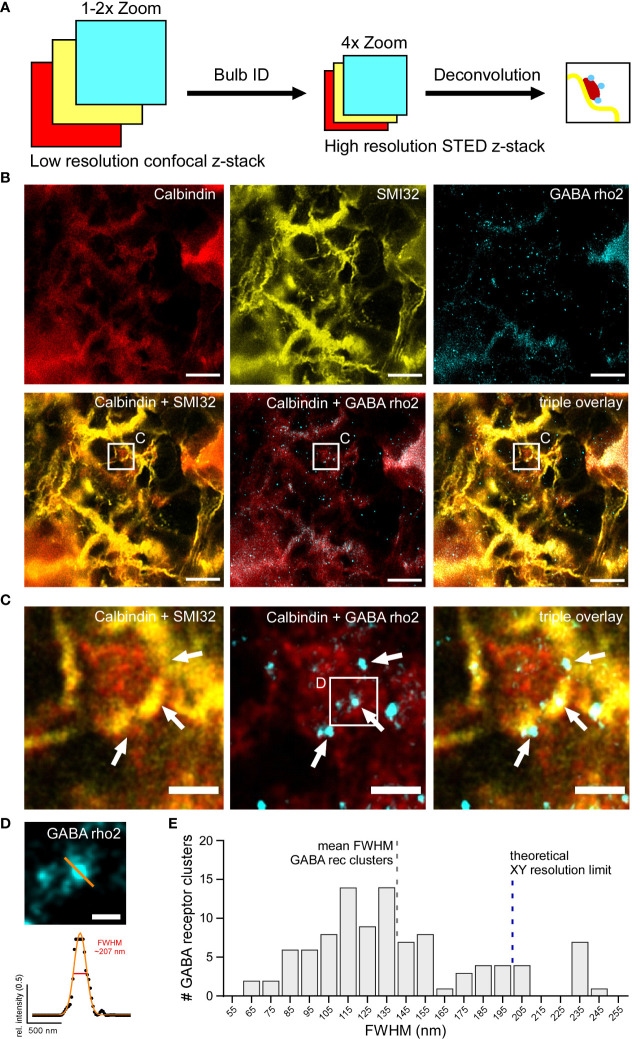
GABA_C_ receptor clusters can be localised on horizontal cell dendritic bulbs with high-resolution STED **(A)** Schematic showing experimental design to image dendritic HC bulbs with high-resolution STED microscopy. Individual bulbs are identified in sections of large confocal z-stacks (Bulb ID, see also **Figure 3**). Bulbs were zoomed into, imaged as STED z-stacks and visualised using maximum z-projections and deconvolution. **(B)** Triple staining experiment against Calbindin (red), SMI32 (yellow) and GABA ρ2 (cyan) showing a dendritic bulb (white square) imaged in the STED mode. The centre of the bulb is negative for SMI32 but positive for calbindin labelling. White square indicates the position of the bulb depicted as close-ups in C. **(C)** Zoomed-in bulb with triple staining against Calbindin, SMI32 and GABA ρ2 showing a HC dendritic bulb (from white square in B) imaged in STED mode and deconvolved. Note that GABA ρ2 receptor clusters (arrows) are located at the edges of bulb. **(D)** Example ρ2-positive GABA receptor cluster (top, small white square from C) with Gaussian fit and FWHM (bottom). FWHM intensity profile is taken at the indicated position (orange line). **(E)** Histogram showing distribution of FWHMs of GABA ρ2 clusters (in 10 nm bins) (n = 1 animal, n = 100 clusters). Dashed blue bar indicates xy resolution limit for confocal light microscopy (~ 200 nm), dashed grey bar shows FWHM mean for GABA receptor clusters (~ 140 nm, see Results section). For SMI32, the excitation wavelength was 532 nm, and the dye was ATTO532. For Calbindin, the excitation wavelength was 488 nm, and the dye was STAR488. For GABA ρ2, the excitation wavelength was 635 nm, and the dye was ATTO633. Scale bars: 5 µm in B, 1 µm in C, 0.25 µm in D.

To image HC bulbs and GABA ρ2 receptors with higher resolution, STED images of identified bulb structures were acquired. Bulbs were identified as described above using large confocal z-stacks, which were screened for bulbs directly at the microscope (**Figure 8A**). For this triple labelling approach, each primary antibody was paired with multiple secondary antibodies, coupled to different fluorophores, and imaged using both confocal and STED microscopy to identify fluorophores working optimally with three-colour STED (**Figure 8B**). Fluorophores of the Alexa Fluor family were tested but found to easily bleach. In the end, fluorophores of the STAR and ATTO families were chosen for their superior photostability, although in confocal imaging, they can be dimmer compared to Alexa Fluor dyes. To avoid spectral overlap in the triple staining and make use of the available excitation and depletion lasers, STAR488 was chosen for the Calbindin, ATTO532 for SMI32, and ATTO633 for GABA ρ2 staining. Bulbs were then zoomed-in using confocal live-view before Calbindin, SMI32 and GABA ρ2 stainings were subsequently imaged with the STED mode. Deconvolution was applied afterwards to increase SBR and better resolve the fine GABA receptor clusters. Interestingly, bulbs identified in confocal mode were often only weakly visible with higher-magnification (4.0× digital zoom) STED microscopy. However, bulbs that could be observed with STED correlated with multiple GABA receptor blobs/clusters (**Figure 8C**). Finally, we determined the FWHMs of GABA receptor clusters in deconvolved STED images (**Figure 8D**). Our quantification showed that the vast majority (93 out of 100) of analysed GABA ρ2-positive clusters had an FWHM under 200 nm, surpassing the xy resolution limit of confocal light microscopy. In fact, many FWHMs peaked at 110 to 120 nm (140.1 ± 443.8 nm, mean ± SD, n = 100 receptor clusters), which is within the ‘working range’ of the resolution limit of super-resolution STED microscopy (**Figure 8E**).

In conclusion, we were able to reliably identify GABA receptor clusters on HC dendritic bulbs using super-resolution STED microscopy and could show that the FWHM of the GABA receptor clusters is clearly under the resolution limit of confocal microscopy.

## Discussion

Super-resolution imaging of deeper layers of specimens has been established for different brain structures (11, 39). Here, we developed a reliable protocol for STED imaging in the whole-mount mouse retina. So far, the number of STED protocols for mouse retinal tissue is very limited (26). To optimise the imaging procedure, we adapted sample preparation and microscope settings. As the first retinal model system, we labelled neurofilaments in RGCs and found that – at a depth of up to ~30 µm – the spatial xy resolution could be increased by a factor of > 2. Next, we aimed at imaging synaptic structures of HCs in the outer retina at a depth of ~130 µm. Due to scattering of the laser light and misalignment of the excitation and depletion laser beams, super-resolution imaging in deeper layers of the outer retina did not yield resolution improvements. However, we established a method to circumvent this problem and to increase the resolution of STED microscopy when imaging in those deeper retinal layers: horizontal cryosectioning and removal of the inner retinal tissue allowed us to access HC neurites with STED imaging. Here, we visualised dendritic bulbs, which likely represent a novel type of synapse capable of GABAergic feedforward signalling from HCs to BCs. Thus, imaging in the whole-mount retina can help to describe the protein composition and scaffold at retinal synapses. Taken together, we are convinced that our protocol further expands the application portfolio of STED microscopy.

### Resolution increment is determined by the saturation factor of the depletion laser

For single-colour STED microscopy and for imaging the fine structures as GABA receptor clusters, we chose the 775 nm depletion beam because with it we obtained a higher saturation factor than with the 592 nm laser. The two lasers differed in both laser architecture and temporal properties of excitation, depletion and detection. The 775 nm pulsed/gated laser allowed us to achieve a saturation factor of 30, which was theoretically sufficient for resolution scaling up to 6.79 times if compared with confocal microscopy (35). The main constraint with increasing depletion laser intensity was specimen overheating. Another problem that we faced was the loss of SBR with increasing imaging depth and light scattering in the tissue. To our knowledge, it is a common problem with several possible solutions (19, 20). We tried deconvolution and obtained a pronounced SBR improvement. To further increase contrast and improve the axial resolution, one can use a 3D STED approach with additional donut-shaped illumination perpendicularly to the optical axis (40) or adaptive optics (36, 37), or alternatively horizontal cryosectioning (see below).

### Optimal resolution with the conventional antibody approach in the whole-mounted retina

A very crucial factor of every super-resolution microscopy approach is resolution estimation. For this purpose, we fitted a Gaussian to the line profiles of filamentous structures using the NLS algorithm. From the Gaussian fits, we determined the FWHMs and compared the values between imaging conditions. This approach, although widely used (41–43), is prone to errors (19) and relatively laborious. It requires isolated filamentous or punctuated nanometre-sized structures, which are not necessarily easy to find even in the samples labelled against cytoskeletal or calcium-binding proteins. In contrast, another approach, Fourier ring correlation analysis, could be performed without any prior information about the sample (44). Given the abundance of the antigen and the antibodies concentration, we had sufficient labelling density. However, given that the intermediate filament has a width of ~10 nm and is labelled by indirect immunodetection with antibodies having size of ~20 nm, the smallest measurable biological structure is theoretically restricted to approximately 50 nm in our case (45). Furthermore, the labelling with IgG antibodies may introduce artefacts into the imaging (40) making some densely packed antigens inaccessible for labelling. This challenge can be overcome by targeted labelling with small molecules (e.g. nanobodies, protein/peptide-directed labelling, aptamers or click chemistry-based labelling of single amino acids (46–49)).

### Sample preparation and immunolabeling for STED microscopy in the whole-mount mouse retina

In combination with the cryosectioning, a protocol for an immunocytochemistry triple staining for STED imaging was developed and tested in both whole-mount retinae and horizontal retinal cryosections. Antibodies against Calbindin, SMI32, and GABA ρ2 were chosen for assessing HC synapses. However, we expect that the protocol also works with other antibodies. In general, all tested antibody stainings functioned as expected, although problems with the GABA ρ2 antibody such as insufficient penetration of the tissue occurred from time to time. Possible explanations might be a lower affinity of the antibody, or more generally, in the antigen properties of GABA_C_ receptors, whose epitopes may be hidden within the double lipid membrane or beneath other synaptic proteins. Additionally, GABA receptor clusters are smaller and sparser than SMI32 and Calbindin complexes, resulting in a lower overall number of target epitopes. As Calbindin is present throughout the HC cytosol, its staining often appears to be diffuse and HC structures appear blurry, especially with higher magnification. Alternative approaches to stain whole HCs, such as immunostaining against GFP in transgenic animals or direct injection of fluorophores into single HCs, may result in signals with a better SBR (50).

### Retinal cryosectioning is compatible with standard immunocytochemistry and STED super-resolution microscopy

One major challenge of our approach was to show whether cryosectioning of the retina is still compatible with standard triple staining protocols using STED-compatible fluorophores. Here, we demonstrate the feasibility of performing complex super-resolution STED experiments in retinal cryosections. The newly developed protocol was applied to study HC dendritic bulbs, which likely represent a recently identified synapse for GABAergic feedforward signalling from HCs to BCs (33).

As STED microscopy is based on confocal microscopy technology, sharing the pinhole, it is intrinsically capable of optical sectioning, and thus, should be theoretically able to image in deeper planes of thick specimens. However, the spatial resolution of STED microscopy strongly depends on the exact alignment of the depletion laser donut around the excitation laser beam, which can be disturbed by scattering in biological samples. Multiple adaptive optics approaches, which measure and compensate for the distortions for each point, have been developed but involve multiple drawbacks, mainly high costs and increased imaging time and photobleaching. The method described here averts optical distortions by removing biological tissue above the plane of interest. Additionally, it enables the application of conventional STED microscopy without requiring special settings or computations and thus decreases costs, effort, and imaging time compared to adaptive optics. Cryosectioning is a well-established and widely used technique. Still, some problems, including freezing artefacts as well as wrinkled and displaced sections, are commonly reported and could influence tissue integrity and STED resolution (51). Even small artefacts, hardly visible under confocal microscopes, could influence images taken with STED. Thus, precise and meticulous work is important throughout the whole freezing and cryosectioning process.

### Comparing neurofilament structures of retinal ganglion cells and horizontal cells

In this study, we used the FWHM in biological samples as an indicator for the effective resolution. We demonstrated that both the absolute FWHMs as well as the REF of HC structures were significantly enhanced in cryosections compared with the intact retinal whole-mount, and thus, similar to the resolution of superficial RGC axons and dendrites in the whole-mount retinae. It should be emphasised that in our experiments, the FWHM measured for individual RGC dendrites was lower than the FWHM in co-fasciculating HC neurites. Thus, the measured FWHM does not necessarily reflect the absolute theoretical resolution of the microscope but, for instance, depends on the width of the measured structure, on the thickness of the biological specimen, the size/type of the antibodies and the SBR. Still, the FWHM has been used as a reliable indicator for resolution in past studies (52, 53). Furthermore, the relative resolutions, which were subsequently calculated, partially correct for different-sized structures and confirm the effects observed with the absolute FWHMs. The robustness of curve fitting and thus FWHM calculation also strongly depended on the SBR of the structures selected. For example, in some STED images, the SBR was frequently inadequate, and the accuracy of the fitted curves had to be manually reviewed for each structure. Due to the low number of emitted and detected photons, a low SBR is a general issue with STED imaging. Thus, for each experiment appropriate settings must be determined and the right balance between signal and resolution has to be defined. Theoretically, STED microscopy can reach xy resolutions in the low nm range and, using fluorescent beads, PSFs with as little as 5.8 nm width have been recorded (54). However, these resolutions cannot be currently achieved in biological samples and, while proof-of-concept studies could produce resolutions of around 20 nm (55), the measured maximal resolution is in practice often limited by target size, optical distortions, photobleaching, and labelling strength. In this study, FWHMs of below 100 nm, and thus far beyond the diffraction limit of confocal microscopy, were observed. Still, even under best possible STED imaging conditions, the FWHMs of the HC structures were on average above 200 nm. One reason for this could simply be the relatively large size of the neurofilament structures stained in HCs. This possibility is supported by the fact that although STED resolutions in this experiment are beyond the diffraction limit, they still represent a significant improvement compared to the confocal FWHMs of the same structures. Another argument in favour of this view comes from imaging individual and sparse GABA receptor clusters at HC bulbs: here, the mean FWHM is around 140 nm, and therefore, is close to the mean FWHM of RGC neurofilaments.

Huygens deconvolution was applied to STED images to further increase the resolution and improve the SBR. Deconvolution algorithms are computational methods that try to recalculate the original optical scene in the sample by subtracting effects of known optical distortions and diffractions from the image (56) and that were used with STED microscopy before with excellent results (57, 58). In our case however, only minor improvements of FWHM could be observed after deconvolution, although SBR mostly appeared to be increased. A problem with the employed deconvolution algorithm might be that it is “blind”, meaning that it uses a theoretical PSF that was calculated for the microscope setup present. A better approach would include measuring real PSFs using the exact imaging conditions, which might enable the deconvolution to predict optical aberrations more accurately.

### Horizontal cell bulbs likely represent GABAergic presynapses

Horizontal cells are essential for the generation of centre-surround receptive fields in BCs (59). While most studies focused on the complex HC feedback mechanisms to photoreceptors, evidence for direct HC-to-BC synaptic contacts has been found in both non-mammalian and mammalian retinae (31, 32, 60–62). HC feedforward signalling is likely GABAergic and dependent on vesicular release (63, 64). Recently, bulbs on HC dendrites have been observed in 3D electron microscopy reconstructions and identified as possible synaptic contacts, with most bulbs contacting either other HC bulbs or BC dendrites (33). Furthermore, Behrens and colleagues demonstrated the presence of mitochondria in dendritic bulbs and used immunolabeling to reveal that bulbs of individually stained horizontal cells co-localize GABA ρ2 receptors. In this study, bulb identification was performed by using Calbindin as a marker for the entire HC and additionally labelling SMI32 to counterstain HC axon terminals. This allowed the distinction between dendrites and axons and by calculating the intensity differences between Calbindin and SMI32 signals quantitative characteristics of bulbs could be defined. We originally expected to find half of the HC structures double-positive, representing axons, and the other half only positive for Calbindin, representing dendrites. However, very few Calbindin-only-positive structures could be observed. Space limitations in the outer plexiform layer and a high density of HC structures likely result in strong co-fasciculation of HC dendrites and axons and an overlay of single- and double-positive structures. Still, by imaging large z-stacks with low magnification in the confocal mode, single bulb structures were frequently identified. These structures typically emerged from double-positive filamentous structures and occasionally overlapped with additional SMI32 signals, which might be the result of HC axons stratifying along the bulbs. Nevertheless, these structures showed no correlation between Calbindin and SMI32 signals and were thus further considered dendritic bulbs.

By detecting the ρ2 subunit of GABA_C_ receptors, which in the retina are exclusively expressed on BCs (65), at Calbindin-positive/SMI32-negative bulbs, we could specifically investigate the role of bulbs in HC to BC GABAergic signalling. Some GABA ρ2 signals were detected on or in direct proximity to bulbs, thus indicating co-localization of bulbs with BC postsynaptic sites, and supporting the notion that bulbs are HC-to-BC synapses. Still, the presence of additional synaptic markers (e.g. presynaptic proteins) would provide further evidence for the type and mechanism of bulb synapses. For instance, synaptic proteins of the release machinery in HC dendrites as well as GABA and GAD65 have been found in mammalian HCs (33, 64, 66). It might be compelling to monitor these targets with super-resolution microscopy and compare their localization within bulbs to the GABA receptors. Further intriguing imaging targets are voltage-gated calcium channels, although antibodies against them are either extremely subtype-specific and have a low efficiency or are very unspecific, resulting in cross-reactions with other targets (67). In general, staining as many targets simultaneously as possible would enable the extraction of much more information about the synapse ultrastructure, however, immunocytochemistry gets increasingly difficult the more simultaneous stainings are applied and chances for unspecific bindings or fluorescent crosstalk grow. Functional experiments unravelling the mechanisms and function of bulb synapses would be highly desirable, but selectively monitoring or manipulating HC-to-BC synapses is challenging due to the complexity of outer retinal circuits (e.g. simultaneous feedback and feedforward signalling within a single interneuron) and the presence of similarly complex synapses between photoreceptors, BCs and HCs in close proximity. Nonetheless, structural super-resolution studies such as the present work might provide novel access points for further experiments.

## Data availability statement

The raw data supporting the conclusions of this article will be made available by the authors, without undue reservation.

## Ethics statement

The animal study was reviewed and approved by Tierschutzbeauftragte der Universität Tübingen.

## Author contributions

LK, KS: Conceptualisation, investigation, methodology, data analysis, writing (original draft), visualisation. TS: Conceptualisation, investigation, writing (original draft), visualisation, supervision. IN-S: Conceptualisation, visualisation, supervision, writing (review and editing). FH: writing (review and editing), visualization. TE: writing (review and editing), visualisation, supervision. MU: writing (review and editing), visualisation, funding acquisition. ZZ: investigation, data analysis, visualisation, writing (review and editing). All authors contributed to the article and approved the submitted version. 

## References

[1] Mishina NM Mishin AS Belyaev Y Bogdanova EA Lukyanov S Schultz C . Live-cell STED microscopy with genetically encoded biosensor. Nano Lett (2015) 15(5):2928–32. doi: 10.1021/nl504710z 25871892

[2] Chojnacki J Staudt T Glass B Bingen P Engelhardt J Anders M . Maturation-dependent HIV-1 surface protein redistribution revealed by fluorescence nanoscopy. Science (2012) 338(6106):524–8. doi: 10.1126/science.1226359 23112332

[3] Ratz M Testa I Hell SW Jakobs S . CRISPR/Cas9-mediated endogenous protein tagging for RESOLFT super-resolution microscopy of living human cells. Sci Rep (2015) 5:9592. doi: 10.1038/srep09592 25892259 PMC4402611

[4] Hell SW Wichmann J . Breaking the diffraction resolution limit by stimulated emission: stimulated-emission-depletion fluorescence microscopy. Opt Lett (1994) 19(11):780–2. doi: 10.1364/OL.19.000780 19844443

[5] Maglione M Sigrist SJ . Seeing the forest tree by tree: super-resolution light microscopy meets the neurosciences. Nat Neurosci (2013) 16(7):790–7. doi: 10.1038/nn.3403 23799471

[6] D’Este E Kamin D Balzarotti F Hell SW . Ultrastructural anatomy of nodes of ranvier in the peripheral nervous system as revealed by STED microscopy. Proc Natl Acad Sci U S A (2017) 114(2):E191–9. doi: 10.1073/pnas.1619553114 PMC5240729 28003466

[7] Kellner RR Baier CJ Willig KI Hell SW Barrantes FJ . Nanoscale organization of nicotinic acetylcholine receptors revealed by stimulated emission depletion microscopy. Neuroscience (2007) 144(1):135–43. doi: 10.1016/j.neuroscience.2006.08.071 17049171

[8] Tang AH Chen H Li TP Metzbower SR MacGillavry HD Blanpied TA . A trans-synaptic nanocolumn aligns neurotransmitter release to receptors. Nature (2016) 536(7615):210–4. doi: 10.1038/nature19058 PMC5002394 27462810

[9] Kempf C Staudt T Bingen P Horstmann H Engelhardt J Hell SW . Tissue multicolor STED nanoscopy of presynaptic proteins in the calyx of held. PloS One (2013) 8(4):e62893. doi: 10.1371/journal.pone.0062893 23658655 PMC3637247

[10] Nishimune H Badawi Y Mori S Shigemoto K . Dual-color STED microscopy reveals a sandwich structure of bassoon and piccolo in active zones of adult and aged mice. Sci Rep (2016) 6:27935. doi: 10.1038/srep27935 27321892 PMC4913281

[11] Urban NT Willig KI Hell SW Nägerl UV . STED nanoscopy of actin dynamics in synapses deep inside living brain slices. Biophys J (2011) 101(5):1277–84. doi: 10.1016/j.bpj.2011.07.027 PMC3164186 21889466

[12] Willig KI Steffens H Gregor C Herholt A Rossner MJ Hell SW . Nanoscopy of filamentous actin in cortical dendrites of a living mouse. Biophys J (2014) 106(1):L01–3. doi: 10.1016/j.bpj.2013.11.1119 PMC3907229 24411266

[13] Fuhrmann M Gockel N Arizono M Dembitskaya Y Nägerl UV Pennacchietti F . Super-resolution microscopy opens new doors to life at the nanoscale. J Neurosci (2022) 42(45):8488–97. doi: 10.1523/JNEUROSCI.1125-22.2022 PMC9665916 36351828

[14] Rust MJ Bates M Zhuang X . Sub-Diffraction-limit imaging by stochastic optical reconstruction microscopy (STORM). Nat Methods (2006) 3(10):793–5. doi: 10.1038/nmeth929 PMC2700296 16896339

[15] Betzig E Patterson GH Sougrat R Lindwasser OW Olenych S Bonifacino JS . Imaging intracellular fluorescent proteins at nanometer resolution. Science (2006) 313(5793):1642–5. doi: 10.1126/science.1127344 16902090

[16] Hess ST Girirajan TPK Mason MD . Ultra-high resolution imaging by fluorescence photoactivation localization microscopy. Biophys J (2006) 91(11):4258–72. doi: 10.1529/biophysj.106.091116 PMC1635685 16980368

[17] Heilemann M van de Linde S Schüttpelz M Kasper R Seefeldt B Mukherjee A . Subdiffraction-resolution fluorescence imaging with conventional fluorescent probes. Angewandte Chemie Int Edition (2008) 47(33):6172–6. doi: 10.1002/anie.200802376 18646237

[18] Tam J Merino D . Stochastic optical reconstruction microscopy (STORM) in comparison with stimulated emission depletion (STED) and other imaging methods. J Neurochem (2015) 135(4):643–58. doi: 10.1111/jnc.13257 26222552

[19] Lambert TJ Waters JC . Navigating challenges in the application of superresolution microscopy. J Cell Biol (2017) 216(1):53–63. doi: 10.1083/jcb.201610011 27920217 PMC5223610

[20] Vicidomini G Bianchini P Diaspro A . STED super-resolved microscopy. Nat Methods (2018) 15(3):173–82. doi: 10.1038/nmeth.4593 29377014

[21] Baier H . Synaptic laminae in the visual system: molecular mechanisms forming layers of perception. Annu Rev Cell Dev Biol (2013) 29:385–416. doi: 10.1146/annurev-cellbio-101011-155748 24099086

[22] Chávez AE Singer JH Diamond JS . Fast neurotransmitter release triggered by Ca influx through AMPA-type glutamate receptors. Nature (2006) 443(7112):705–8. doi: 10.1038/nature05123 17036006

[23] Baden T Euler T Weckström M Lagnado L . Spikes and ribbon synapses in early vision. Trends Neurosci (2013) 36(8):480–8. doi: 10.1016/j.tins.2013.04.006 23706152

[24] Nath A Schwartz GW . Electrical synapses convey orientation selectivity in the mouse retina. Nat Commun (2017) 8(1):2025. doi: 10.1038/s41467-017-01980-9 29229967 PMC5725423

[25] Lv C Gould TJ Bewersdorf J Zenisek D . High-resolution optical imaging of zebrafish larval ribbon synapse protein RIBEYE, RIM2, and CaV 1.4 by stimulation emission depletion microscopy. Microsc Microanal (2012) 18(4):745–52. doi: 10.1017/S1431927612000268 PMC3709260 22832038

[26] Schlüter A Rossberger S Dannehl D Janssen JM Vorwald S Hanne J . Dynamic regulation of synaptopodin and the axon initial segment in retinal ganglion cells during postnatal development. Front Cell Neurosci (2019) 13:318. doi: 10.3389/fncel.2019.00318 31417359 PMC6682679

[27] Kerschensteiner D . Feature detection by retinal ganglion cells. Annu Rev Vis Sci (2022) 8:135–69. doi: 10.1146/annurev-vision-100419-112009 35385673

[28] Kemmler R Schultz K Dedek K Euler T Schubert T . Differential regulation of cone calcium signals by different horizontal cell feedback mechanisms in the mouse retina. J Neurosci (2014) 34(35):11826–43. doi: 10.1523/JNEUROSCI.0272-14.2014 PMC6608415 25164677

[29] Dowling JE Brown JE Major D . Synapses of horizontal cells in rabbit and cat retinas. Science (1966) 153(3744):1639–41. doi: 10.1126/science.153.3744.1639 5917075

[30] Dowling JE . Synaptic organization of the frog retina: an electron microscopic analysis comparing the retinas of frogs and primates. Proc R Soc Lond B Biol Sci (1968) 170(1019):205–28. doi: 10.1098/rspb.1968.0034 4385244

[31] Kolb H Jones J . Synaptic organization of the outer plexiform layer of the turtle retina: an electron microscope study of serial sections. J Neurocytol (1984) 13(4):567–91. doi: 10.1007/BF01148080 6481412

[32] Linberg KA Fisher SK . Ultrastructural evidence that horizontal cell axon terminals are presynaptic in the human retina. J Comp Neurol (1988) 268(2):281–97. doi: 10.1002/cne.902680211 3360989

[33] Behrens C Yadav SC Korympidou MM Zhang Y Haverkamp S Irsen S . Retinal horizontal cells use different synaptic sites for global feedforward and local feedback signaling. Curr Biol (2022) 32(3):545–58.e5. doi: 10.1016/j.cub.2021.11.055 34910950 PMC8886496

[34] Chapot CA Euler T Schubert T . How do horizontal cells “talk” to cone photoreceptors? different levels of complexity at the cone-horizontal cell synapse. J Physiol (2017) 595(16):5495–506. doi: 10.1113/JP274177 PMC5556172 28378516

[35] Harke B Keller J Ullal CK Westphal V Schönle A Hell SW . Resolution scaling in STED microscopy. Opt Express (2008) 16(6):4154–62. doi: 10.1364/OE.16.004154 18542512

[36] Hao X Allgeyer ES Lee DR Antonello J Watters K Gerdes JA . Three-dimensional adaptive optical nanoscopy for thick specimen imaging at sub-50-nm resolution. Nat Methods (2021) 18(6):688–93. doi: 10.1038/s41592-021-01149-9 PMC7610943 34059828

[37] Gould TJ Burke D Bewersdorf J Booth MJ . Adaptive optics enables 3D STED microscopy in aberrating specimens. Opt Express (2012) 20(19):20998–1009. doi: 10.1364/OE.20.020998 PMC3635694 23037223

[38] Peichl L González-Soriano J . Unexpected presence of neurofilaments in axon-bearing horizontal cells of the mammalian retina. J Neurosci (1993) 13(9):4091–100. doi: 10.1523/JNEUROSCI.13-09-04091.1993 PMC6576459 8366362

[39] Nägerl UV Willig KI Hein B Hell SW Bonhoeffer T . Live-cell imaging of dendritic spines by STED microscopy. Proc Natl Acad Sci U S A (2008) 105(48):18982–7. doi: 10.1073/pnas.0810028105 PMC2585941 19028874

[40] Fernández-Suárez M Ting AY . Fluorescent probes for super-resolution imaging in living cells. Nat Rev Mol Cell Biol (2008) 9(12):929–43. doi: 10.1038/nrm2531 19002208

[41] Lukinavičius G Mitronova GY Schnorrenberg S Butkevich AN Barthel H Belov VN . Fluorescent dyes and probes for super-resolution microscopy of microtubules and tracheoles in living cells and tissues. Chem Sci (2018) 9(13):3324–34. doi: 10.1039/C7SC05334G PMC5932598 29780462

[42] Schnorrenberg S Grotjohann T Vorbrüggen G Herzig A Hell SW Jakobs S . *In vivo* super-resolution RESOLFT microscopy of drosophila melanogaster. eLife (2016) 5:e15567. doi: 10.7554/eLife.15567 27355614 PMC4927295

[43] Pellett PA Sun X Gould TJ Rothman JE Xu MQ Corrêa IR Jr . Two-color STED microscopy in living cells. BioMed Opt Express (2011) 2(8):2364–71. doi: 10.1364/BOE.2.002364 PMC3149534 21833373

[44] Tortarolo G Castello M Diaspro A Koho S . Evaluating image resolution in stimulated emission depletion microscopy. Optica (2018) 5:32–5. doi: 10.1364/OPTICA.5.000032

[45] Görlitz F Hoyer P Falk H Kastrup L Engelhardt J Hell SW . A STED microscope designed for routine biomedical applications. Prog Electromagn Res B Pier B (2014) 147:57–68. doi: 10.2528/PIER14042708

[46] Hein B Willig KI Wurm CA Westphal V Jakobs S Hell SW . Stimulated emission depletion nanoscopy of living cells using SNAP-tag fusion proteins. Biophys J (2010) 98(1):158–63. doi: 10.1016/j.bpj.2009.09.053 PMC2800968 20074516

[47] Arsić A Hagemann C Stajković N Schubert T Nikić-Spiegel I . Minimal genetically encoded tags for fluorescent protein labeling in living neurons. Nat Commun (2022) 13(1):314. doi: 10.1038/s41467-022-27956-y 35031604 PMC8760255

[48] Reshetniak S Rizzoli SO . Interrogating synaptic architecture: Approaches for labeling organelles and cytoskeleton components. Front Synaptic Neurosci (2019) 11:23. doi: 10.3389/fnsyn.2019.00023 31507402 PMC6716447

[49] Opazo F Levy M Byrom M Schäfer C Geisler C Groemer TW . Aptamers as potential tools for super-resolution microscopy. Nat Methods (2012) 9(10):938–9. doi: 10.1038/nmeth.2179 23018995

[50] Schubert T Huckfeldt RM Parker E Campbell JE Wong ROL . Assembly of the outer retina in the absence of GABA synthesis in horizontal cells. Neural Dev (2010) 5:15. doi: 10.1186/1749-8104-5-15 20565821 PMC2919532

[51] Watkins S . Cryosectioning. Curr Protoc Mol Biol (2001), 1–8. doi: 10.1002/0471142727.mb1402s07 18265110

[52] Klar TA Jakobs S Dyba M Egner A Hell SW . Fluorescence microscopy with diffraction resolution barrier broken by stimulated emission. Proc Natl Acad Sci U S A (2000) 97(15):8206–10. doi: 10.1073/pnas.97.15.8206 PMC26924 10899992

[53] Dzyubenko E Rozenberg A Hermann DM Faissner A . Colocalization of synapse marker proteins evaluated by STED-microscopy reveals patterns of neuronal synapse distribution in vitro. J Neurosci Methods (2016) 273:149–59. doi: 10.1016/j.jneumeth.2016.09.001 27615741

[54] Rittweger E Han KY Irvine SE Eggeling C Hell SW . STED microscopy reveals crystal colour centres with nanometric resolution. Nat Photonics (2009) 3(3):144–7. doi: 10.1038/nphoton.2009.2

[55] Wildanger D Medda R Kastrup L Hell SW . A compact STED microscope providing 3D nanoscale resolution. J Microsc (2009) 236(1):35–43. doi: 10.1111/j.1365-2818.2009.03188.x 19772534

[56] Sibarita JB . Deconvolution microscopy. Adv Biochem Eng Biotechnol (2005) 95:201–43. doi: 10.1007/b102215 16080270

[57] Willig KI Keller J Bossi M Hell SW . STED microscopy resolves nanoparticle assemblies. New J Physics (2006) 8(6):106–6. doi: 10.1088/1367-2630/8/6/106

[58] Zanella R Zanghirati G Cavicchioli R Zanni L Boccacci P Bertero M . Towards real-time image deconvolution: application to confocal and STED microscopy. Sci Rep (2013) 3:2523. doi: 10.1038/srep02523 23982127 PMC3755287

[59] Diamond JS . Inhibitory interneurons in the retina: Types, circuitry, and function. Annu Rev Vis Sci (2017) 3:1–24. doi: 10.1146/annurev-vision-102016-061345 28617659

[60] Dowling JE Boycott BB . Organization of the primate retina: electron microscopy. Proc R Soc Lond B Biol Sci (1966) 166(1002):80–111. doi: 10.1098/rspb.1966.0086 4382694

[61] Fisher SK Boycott BB . Synaptic connections made by horizontal cells within the outer plexiform layer of the retina of the cat and the rabbit. Proc R Soc Lond B Biol Sci (1974) 186(1085):317–31. doi: 10.1098/rspb.1974.0052 4154444

[62] Sakai HM Naka K . Synaptic organization of the cone horizontal cells in the catfish retina. J Comp Neurol (1986) 245(1):107–15. doi: 10.1002/cne.902450108 3958241

[63] Thoreson WB Mangel SC . Lateral interactions in the outer retina. Prog Retin Eye Res (2012) 31(5):407–41. doi: 10.1016/j.preteyeres.2012.04.003 PMC3401171 22580106

[64] Puller C Haverkamp S Neitz M Neitz J . Synaptic elements for GABAergic feed-forward signaling between HII horizontal cells and blue cone bipolar cells are enriched beneath primate s-cones. PloS One (2014) 9(2):e88963. doi: 10.1371/journal.pone.0088963 24586460 PMC3930591

[65] Enz R Brandstätter JH Hartveit E Wässle H Bormann J . Expression of GABA receptor rho 1 and rho 2 subunits in the retina and brain of the rat. Eur J Neurosci (1995) 7(7):1495–501. doi: 10.1111/j.1460-9568.1995.tb01144.x 7551175

[66] Hirano AA Vuong HE Kornmann HL Schietroma C Stella SL Jr Barnes S . Vesicular release of GABA by mammalian horizontal cells mediates inhibitory output to photoreceptors. Front Cell Neurosci (2020) 14:600777. doi: 10.3389/fncel.2020.600777 33335476 PMC7735995

[67] Trimmer JS Rhodes KJ . Localization of voltage-gated ion channels in mammalian brain. Annu Rev Physiol (2004) 66:477–519. doi: 10.1146/annurev.physiol.66.032102.113328 14977411

